# Chemical controls on the propagation rate of fracture in calcite

**DOI:** 10.1038/s41598-018-34355-1

**Published:** 2018-11-07

**Authors:** A. G. Ilgen, W. M. Mook, A. B. Tigges, R. C. Choens, K. Artyushkova, K. L. Jungjohann

**Affiliations:** 1 Sandia National Laboratories, Geochemistry Department, 1515 Eubank SE Mailstop 0754, Albuquerque, NM 87185-0754 United States; 2 Sandia National Laboratories, Nanosystems Synthesis/Analysis Department, Center for Integrated Nanotechnologies, Albuquerque, NM United States; 3 Sandia National Laboratories, Geomechanics Department, 1515 Eubank SE Mailstop 0750, Albuquerque, NM 87185-0750 United States; 4 University of New Mexico, Advanced Materials Laboratory, Albuquerque, NM United States

## Abstract

Calcite (CaCO_3_) is one of the most abundant minerals in the Earth’s crust, and it is susceptible to subcritical chemically-driven fracturing. Understanding chemical processes at individual fracture tips, and how they control the development of fractures and fracture networks in the subsurface, is critical for carbon and nuclear waste storage, resource extraction, and predicting earthquakes. Chemical processes controlling subcritical fracture in calcite are poorly understood. We demonstrate a novel approach to quantify the coupled chemical-mechanical effects on subcritical fracture. The calcite surface was indented using a Vickers-geometry indenter tip, which resulted in repeatable micron-scale fractures propagating from the indent. Individual indented samples were submerged in an array of aqueous fluids and an optical microscope was used to track the fracture growth *in situ*. The fracture propagation rate varied from 1.6 × 10^−8^ m s^−1^ to 2.4 × 10^−10^ m s^−1^. The rate depended on the type of aqueous ligand present, and did not correlate with the measured dissolution rate of calcite or trends in zeta-potential. We postulate that chemical complexation at the fracture tip in calcite controls the growth of subcritical fracture. Previous studies indirectly pointed to the zeta-potential being the most critical factor, while our work indicates that variation in the zeta-potential has a secondary effect.

## Introduction

Fractures and fracture networks in the subsurface control fluid flow and transport, while reactions at the fluid-mineral interfaces can influence the development of fractures. Fracturing of rocks takes place when stress conditions exceed critical values; however, chemical processes at individual fracture tips can lower the failure threshold by reducing the energy needed to propagate a fracture, and in this case fractures develop at stress conditions below measured critical values. The subcritical, chemically-assisted fractures in brittle materials propagate due to breakage of chemical bonds at the atomically-sharp fracture tips. The coupled chemical-mechanical processes at individual fracture tips can control macroscopic features of fractures^1,2^ and fracture networks in the subsurface^3,4^, as well as the time- and scale-dependent deformation of geomaterials^5–9^.

The objective of our experimental work is to develop a mechanistic understanding of the chemical processes controlling subcritical fracture in calcite. We examined fracture growth rate as a function of three chemical variables: (i) calcite dissolution rate; (ii) changes in the zeta-potential at calcite surface; and (iii) chemical complexation at the fracture tip. We developed a novel approach for investigating chemical effects on subcritical fracture by modifying chemistry at preexisting and prestressed fracture tips to induce fracture growth. The (100) surface of synthetic calcite was indented under dry conditions, resulting in repeatable micron-scale fractures propagating from the corners of the indent, along the cleavage planes in calcite. The plastic zone surrounding the indentation site created a residual stress field that controlled the length of the initial fractures. Individual indented samples were then submerged in aqueous fluids with differing compositions and pH, and optical microscopy was used for *in situ* (in liquid) imaging of the indentation area. The initial fractures grew upon exposure to liquids due to chemical processes at the fracture tip loaded by the residual indentation stress field. Our dataset illustrates that molecular-scale chemical complexation processes at the fracture tip control the propagation rate of subcritical fracture in pre-stressed calcite, in the absence of an external mechanical load.

At the fracture tip, Griffith theory states that the internal energy of the system (U) is equal to the sum of the elastic potential energy of chemical bonds at the fracture tip (U_E_), the external work exerted on the system (W_L_), and the energy from the added surface area of the crack (U_S_):

1 $${\boldsymbol{U}}=({{\boldsymbol{U}}}_{{\boldsymbol{E}}}-{{\boldsymbol{W}}}_{{\boldsymbol{L}}})+{{\boldsymbol{U}}}_{{\boldsymbol{S}}}$$

A fracture will propagate when the work on the system exceeds the potential energy at the fracture tip and energy of creating new surface area. The chemical mechanisms controlling subcritical fracture growth were first proposed almost a century ago, and number of possible mechanisms that address reductions in U_E_ or U_S_ have been proposed since that time^6,10–15^. These mechanisms include: (i) *Stress-corrosion cracking*, when stressed chemical bonds at the fracture tips are more reactive to corrosive agents compared to un-strained bonds; (ii) *Diffusion- controlled subcritical fracture*, similar to stress corrosion cracking, but observed at low stress intensities and limited by the transport of reactive species to the crack tip; (iii) *Dissolution-driven fracturing*, where mineral dissolution rate controls subcritical fracture growth; (iv) *Ion-exchange and complex ion embrittlement*, observed when the exchanging ion introduces lattice strain and assists in the propagation of fracture, and complex ion embrittlement is caused by the solvent-surface reaction where the reacted surface layer is more brittle compared to unreacted material; (v) *Joffe’s effect*, when the fracture tip is blunted due to dissolution in corrosive environments, and fracture growth is stopped by at increase in U_E_. U_S_ mechanisms include *Electrostatic and surface energy effects*, including the Rehbinder effect^10^.

Changes in the surface energy due to chemical alteration (e.g., adsorption of ions or hydrolysis at the crack tip) of the mineral surface, can increase or decrease the energy required for fracture propagation. For example, the zeta (ξ) potential model states that material is most susceptible to subcritical fracture near the point of zero charge^13^. These mechanisms were proposed several decades ago; however, there are few quantitative models capable of predicting subcritical fracture in natural minerals, due to structural and compositional complexity with complicated interfacial chemical processes.

Fractures in rocks can propagate through intergranular cement or through mineral grains. Calcite (CaCO_3_) and other carbonate minerals are common grain-cementing phases in sedimentary rocks like sandstones and mudrocks, as well as constituting both grains and cements in carbonate rocks^16^. Because calcite is a major component of sedimentary rocks, the subcritical fracture of this component may dictate the overall mechanical behavior of calcite-cemented rock masses^17^. Calcite has a perfect [10-11] rhombohedral cleavage, and a relatively fast dissolution rate^18^, compared to silicate and oxide phases^19^.

Subcritical fracture in calcite and carbonate rocks has been studied using double torsion^20,21^ and double cantilever beam^6^ tests on calcite single crystals, and compaction and creep experiments on granular calcite. Rostom *et al*., 2013 studied subcritical fracture along the [10-11] cleavage plane using an *in situ* double torsion technique, where fracture propagation and energy release rate were measured in aqueous solutions with varying concentrations of sodium chloride (NaCl) and ammonium chloride (NH_4_Cl). Weakening (faster fracture growth) was recorded when NaCl concentration was below 0.8 M, and strengthening (slower fracture growth) was observed in the range of > 0.8 M NaCl up to 4 M NaCl, and in 4 wt.% NH_4_Cl solutions^21^. Dissolution kinetics and solubility of calcite had a minor effect on subcritical fracture, and the authors proposed that the changes in surface charge and ξ -potential of calcite with changing electrolyte solutions were controlling subcritical fracture growth^21^. Dunning *et al*., 1994, however, ruled out the ξ -potential effects in calcite, and the authors hypothesized that decreases in surface energy and polymerization reactions at the fracture tip controlled subcritical fracture growth. They used *in situ* double cantilever tests and found no correlation between subcritical fracture and the dissolution rate of calcite. Instead, they observed a complex relationship between subcritical fracture and pH. At pH 2 in hydrochloric (0.01 N) and oxalic (0.05 N) acids, calcite was moderately weaker when compared to de-ionized water; at basic pH of 11.4, calcite was significantly weaker in the 0.1 N NaHCO_3_ solution, while strengthening was observed in 0.005 N NaOH solution. The authors proposed that the weakening was due to the formation of Ca—CO_3_ complexes (polymerization) at the fracture tip, similar to the hydrolysis of the Si—O bonds in quartz^22^ and silica glasses^6,23^.

The increasing activity of water lowers the surface energy in calcite single crystals as well as in carbonate rocks^24^. Røyne *et al*., 2011, demonstrated that subcritical fracture is propagated *via* a single and unidentified physical mechanism, independent of water concentration^24^. Similarly, Risnes *et al*., 2005, quantified weakening of chalk as a function of increasing water concentration using triaxial, hydrostatic, and “Brazilian” tests, hypothesizing that the high affinity of water molecules for calcite surfaces increased pore pressure and decreased grain cohesion^25^.

Spectroscopic surface analysis shows that a freshly fractured calcite surface retains its crystalline structure with a slight re-structuring when exposed to water (even water vapor in the air)^26^. There is also evidence that surface hydrolysis products >Ca—OH and >CO_3_—H form when OH^−^ and H^+^ react with the surface to satisfy the new dangling bonds at the cleavage plane^27^.

Calcite compaction experiments also shed light on the chemical-mechanical coupling. One set of compactions conducted at room temperature illustrate mechanical weakening in the presence of 0.1–0.5 M NaCl, where the compaction rate was three times higher when compared to control experiments with de-ionized water. Additionally, compaction creep was observed in the experiment where the fluid was saturated with respect to CaCO_3_^28^. At 80 °C the presence of 0.6–1 M NaCl increased the compaction rate of calcite, indicating mechanical weakening, while subcritical crack growth increased as NaCl concentration increased to 2–3 M. Creep rates rose even further in the presence of dissolved carbon dioxide (CO_2_)^29^.

Here we show that chemical complexation reactions at the fracture tip exert the primary control on the propagation of subcritical fracture in calcite. We also validate a novel experimental approach for studying subcritical fracture.

## Results and Discussion

### Dissolution rate of calcite

The dissolution rates for calcite were measured on the (100) surface of intact single crystal in the same aqueous solutions as the fracture propagation experiments. The summary of the measured dissolution rates is shown in Table 1. Our measured rates agree with the earlier publications for pH values of 5 and 6, and dissolution rates increase with decreasing pH. The rates at pH values between 2 and 4 are about one order of magnitude lower than rates summarized in the review by Arvidson *et al*.^18^ (Supporting Information). These slower rates are likely due to our measurements being performed on a single crystal (100) surface, compared to previous studies that employed crushed calcite samples. A single crystal surface has fewer defect sites, compared to the crushed powder form, and as a result, a slower dissolution rate. In agreement with previous work^30^, the dissolution rates were weakly affected by the variation in solution ligand.

**Table 1:** Dissolution rates measured on (100) calcite surface, and measured fracture propagation rates (quantified for the linear region from 0 to 15–45 minutes of fracture propagation experiment). The standard deviation for the dissolution rate measurement was 2.0 × 10^−11^ mol cm^−2^ s^−1^, and the standard deviation for the fracture propagation rate was 5.6 × 10^−9^ m s^−1^.

Fluid	pH	Dissolution rate mol cm^−2^ s^−1^	Fracture propagation m s^−1^	Fluid	pH	Dissolution rate mol cm^−2^ s^−1^	Fracture propagation m s^−1^
DI H_2_O	6.5	4.4 × 10^−11^	1.6 × 10^−8^	H_2_C_2_O_4_	5.3	6.0 × 10^−11^	6.3 × 10^−10^
		6.3 × 10^−11^	5.3 × 10^−9^				
		4.2 × 10^−11^	7.6 × 10^−9^				
		5.3 × 10^−11^					
		5.7 × 10^−11^					
		6.6 × 10^−11^					
HCl	4.2	4.7 × 10^−11^	2.3 × 10^−9^	H_2_C_2_O_4_	3.9	1.1 × 10^−10^	9.0 × 10^−9^
			0.8 × 10^−9^			1.2 × 10^−10^	4.8 × 10^−9^
						1.0 × 10^−10^	2.0 × 10^−9^
HCl	3.8	7.1 × 10^−11^	5.2 × 10^−9^	H_2_C_2_O_4_	3.1	3.1 × 10^−10^	
		7.8 × 10^−11^					
		8.5 × 10^−11^					
		9.4 × 10^−11^					
		1.0 × 10^−10^					
		1.1 × 10^−10^					
HCl	3.1	5.9 × 10^−10^	8.5 × 10^−10^	FF	5.7	6.1 × 10^−11^	3.0 × 10^−8^
HCl	2.1	8.8 × 10^−9^	1.7 × 10^−9^	FF	5.0	9.3 × 10^−11^	1.6 × 10^−8^
							9.5 × 10^−9^
H_2_SO_4_	4.5	8.0 × 10^−11^	2.4 × 10^−10^	FF	4.1	2.0 × 10^−10^	1.7 × 10^−8^
		6.2 × 10^−11^				1.8 × 10^−10^	1.7 × 10^−8^
		7.8 × 10^−11^				1.8 × 10^−10^	
						4.4 × 10^−10^	
						3.9 × 10^−10^	
						3.2 × 10^−10^	
H_2_SO_4_	3.8	1.4 × 10^−10^	2.9 × 10^−9^	FF	3.0	9.3 × 10^−10^	8.2 × 10^−10^
H_2_SO_4_	3.0	6.9E × 10^−10^	2.0 × 10^−9^	FF	2.1	2.4 × 10^−9^	2.4 × 10^−9^

### *In situ* fracture growth and fracture toughness

**Figure 1:**
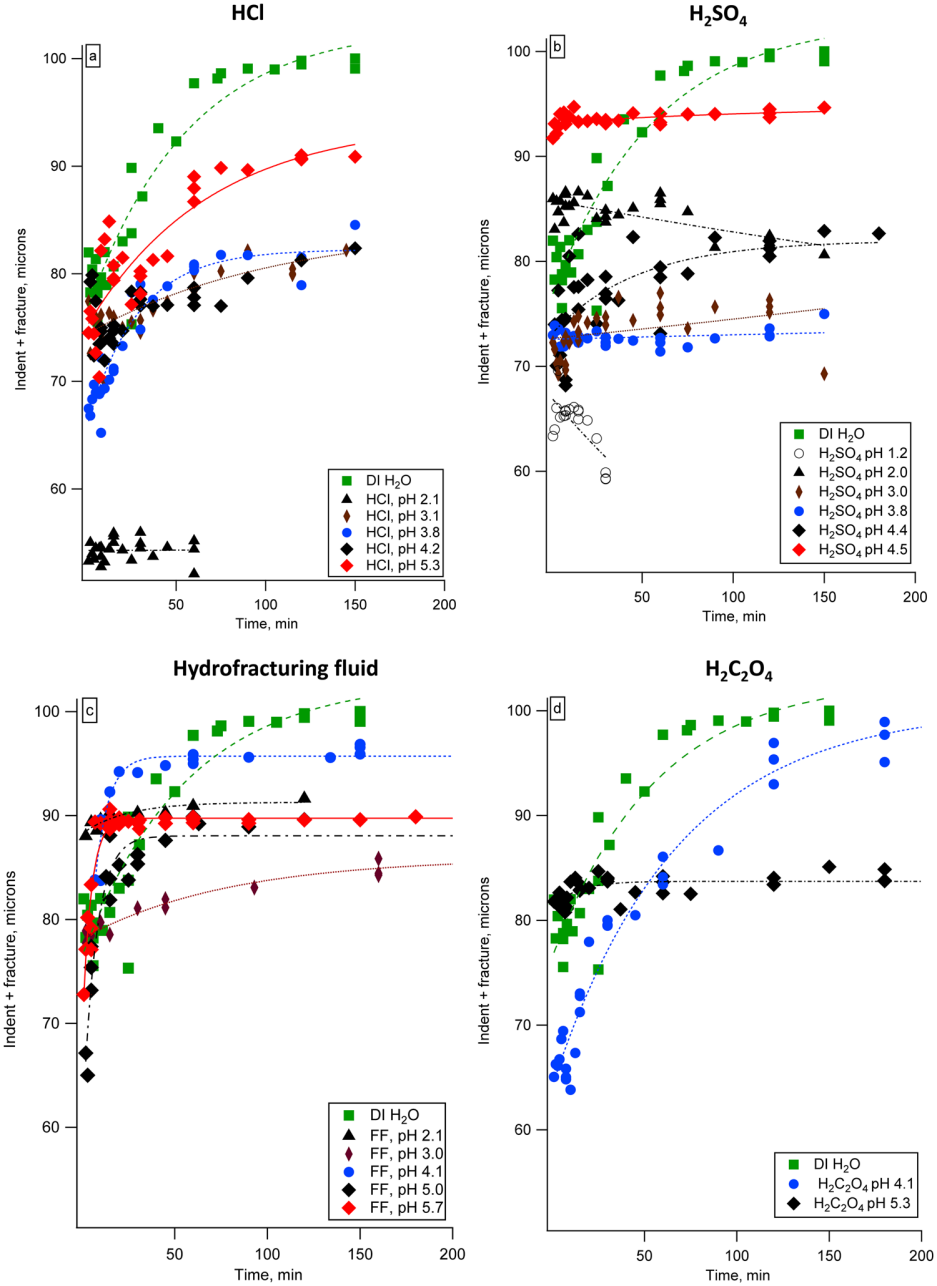
The rates of fracture propagation in calcite: (**a**) in dilute hydrochloric acid, (**b**) in dilute sulfuric acid, (**c**) in synthetic hydrofracturing fluid, and (**d**) in dilute oxalic acid. Fracture growth data for DI H_2_O (green filled squares) is plotted with every series of experiments. Lines show exponential fits. Standard deviation of measurement was on average ±1.5 µm. Error bars are not shown for clarity.

The rates of fracture propagation in calcite submerged in dilute hydrochloric, sulfuric, oxalic acids, and synthetic hydrofracturing fluid are shown in Fig. 1. Fracture propagation rates were non-linear during the 3-hour experiments; the fastest rate was observed soon after exposure to fluids began, and then the fracture propagation rate was linear for 15–45 minutes, depending on the system. Our samples were not under external loading during the fracture propagation measurements, and fracture growth was induced by residual stress at the indentation sites. As the crack extends (moves further from the indent), the magnitude of the residual stress field decreases, and the propagation rate decreases as well. The rates of fracture growth in the linear regions (up to 15–45 minutes following immersion into fluid) were quantified and are shown in Table 1. These measured fracture propagation rates did not correlate with either the calcite dissolution rates nor with the fluid pH. Only synthetic hydrofracturing fluid displayed a positive correlation between fracture propagation rate and pH (Supporting Information). The fracture growth rate measured in our experiments varied from 1.6 × 10^−8^ m s^−1^ to 2.4 × 10^−10^ m s^−1^ (Table 1). Earlier reported values for subcritical fracture in calcite range from 10^−8^ to 10^−4^ m s^−1^, based on double-torsion testing^21^, and for carbonate rocks they range from 10^−9^ to 10^−10^ m s^−1^, based on calcite dissolution rate at the crack tip^12^. Dunning *et al*., 1994 states that the effect of chemical environment is most pronounced for slow-moving fractures close to the stress corrosion limit^6^. Therefore, our experimental setup is well-suited for quantifying chemical effects on subcritical fracture^6^.

The initial cracks exist in equilibrium with the residual stress around the crack tip^31^. The observed crack extension upon exposure to liquids implies a decrease in fracture toughness due to a reduction in U_E_ or U_S_.

The goal of our experimental work was to quantify the decrease in fracture toughness due to fluid exposure, to determine the role of fluid chemistry. To estimate the decrease in fracture toughness, we used the method described by Lawn and Cook, 2012. The fracture toughness can be estimated from^31^:

2 $$\frac{P}{{c}^{3/2}}=\frac{1}{\xi }\times {(\frac{H}{E})}^{1/2}\times T$$

Where *P* is the maximum load (400 mN), *c* is fracture length, measured form the center of the indent, *H* is hardness, *E* is indentation modulus, ξ is the dimensionless constant (0.016 for the Vickers tip), and *T* is fracture toughness. Using our indentation data, we determined that *H* was 3.123 GPa, and *E* was 73.961 GPa. The calculation of fracture toughness is shown in Supporting Information. The estimated fracture toughness prior to *in situ* fracture growth experiment was 0.10–0.16 MPa m^1/2^, and the fracture toughness at the end of the fracture growth experiment decreased by 0.01–0.05 units (Supporting Information). The uncertainty in fracture toughness values is ± 0.01–0.02 MPa m^1/2^, calculated at 26 (95% confidence level).

**Figure 2:**
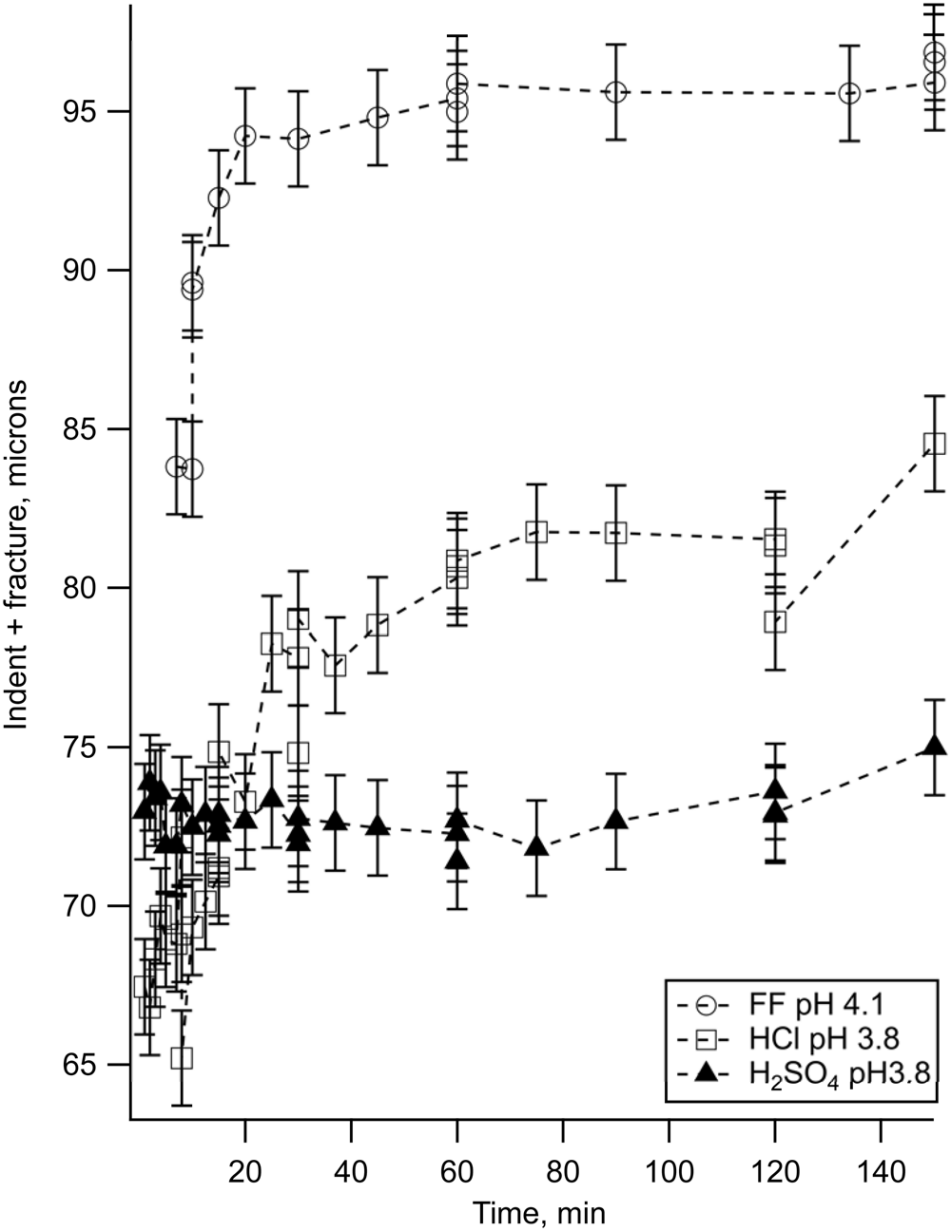
The rates of fracture propagation in calcite in dilute hydrochloric and sulfuric, acids, and in synthetic hydrofracturing fluid at pH ~ 4. Standard deviation of each measurement was ± 1.5 µm (shown).

**Figure 3:**
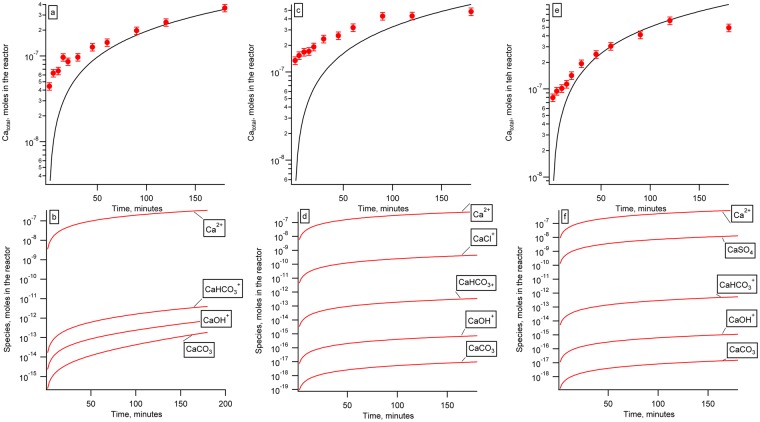
Geochemical modeling results. Measured Ca^2+^ concentrations are shown as filled circles (%RSD = 10), and geochemical models are shown as lines. (**a**) DI H_2_O reactor, data and model; (**b**) calculated aqueous speciation in the DI H_2_O reactor; (**c**) HCl at pH 3.8 reactor data and model; (**d**) calculated aqueous speciation in the HCl at pH 3.8; (**e**) H_2_SO_4_ at pH 3.8 reactor data and model; (**f**) calculated aqueous speciation in the H_2_SO_4_ at pH 3.8.

We observed that fracture propagation rates differed in different aqueous solutions despite similar pH values (e.g. pH ~ 4, shown in Fig. 2) and dissolution rates (Table 1). The fastest fracture propagation was observed in DI water and semi-neutral pH synthetic hydrofracturing fluid (Table 1). To explain the different chemistry effects, we compared our observed fracture rates with the predicted aqueous speciation of calcium in the reactors with DI H_2_O, dilute HCl (pH 3.8), and dilute H_2_SO_4_ (pH 3.8). We used Geochemist’s Workbench software^32^ to calculate aqueous speciation in the reactors. Our reaction path models and corresponding aqueous speciation indicate that with increasing favorability for the Ca-ligand complex (K_β_ for CaCO_3_ is 10^−7.128^; K_β_ for CaCl^+^ is 10^0.7^; and K_β_ for CaSO_4_ is 10^2.32^), fracture growth rate decreases (Fig. 3).

We also calculated aqueous speciation in the solutions used in double torsion experiments by Rostom *et al*., 2012. In this study strengthening was observed in the 0.8 M to 4 M NaCl solutions compared to dilute 0.01 M NaCl solution^21^. Our aqueous speciation calculations show that the most common aqueous species was Ca^2+^, similar to our systems (Supporting Information). However, the ratio of Ca^2+^ to the next most common species—CaCl^+^—increased with increasing NaCl concentration. We propose, that strengthening in the double torsion experiments was observed due to increasing Ca-chloride complexation at the fracture tip, with concentrated NaCl solutions having more chloride ions for Ca-chloride complexation.

### Ex situ characterization of fracture

Profile depth, indent volume, indent width, surface roughness, and average width of the fracture tips are shown in Table 2. For the reacted samples, the indentation sites were a few microns deep, with the deepest being 16 μm. The width of the indent sites in the reacted samples ranged from 13 to 26 μm. The indentation volume was 147 to 208 μm^3^ for unreacted indented calcite samples, and volumes ranged from 273 μm^3^ to 3,472 μm^3^ for reacted samples. Surface roughness was less than 1 nm for most samples, and average fracture width on the surface of the samples was a few microns wide. Like the white light profilometry measurements, confocal Raman indicated that the width of the crack in the control (unreacted) sample is ~ 0.5 microns, measured at 1–2-micron depth, while that for the reacted sample is between 2 to 5 microns at the same depth (Supporting Information). We anticipate that with increasing depth below the sample surface, the fracture tips were atomically sharp, as fractures were growing along the brittle cleavage planes of calcite. However, due to limitations of our measurements, the fracture geometry could not be assessed in 3D below the surface.

**Table 2:** Summary of calcite surface morphology measurements by white light profilometry (measurement schematic is shown in the Methods section).

Sample/Reaction time	Profile Depth (µm)	Indent Volume (µm^3^)	Indent Width (µm)	Surface Roughness (nm)	Fracture Width^a^ (µm)
Unreacted	2.87	208	20.2	0.252	3.09
Unreacted	2.88	147	17.9	0.479	2.74
HCl pH 2.1/60 min	^b^	^b^	^b^	^b^	^b^
HCl pH 3.8/120 min	3.92	1394	16.70	0.93	5.44
HCl pH 4.2/150 min	4.40	303	15.60	0.35	3.68
HCl pH 5.3/150 min	3.24	269	19.40	0.12	2.66
FF pH 2.1/615 min	^b^	^b^	^b^	^b^	^b^
FF pH 3.0/440 min	6.85	2871	25.70	0.50	8.24
FF pH 4.1/180 min	3.50	273	13.10	0.26	4.44
FF pH 5.0/150 min	4.37	295	16.70	0.24	3.98
FF pH 5.0/150 min	3.71	293	17.40	0.35	3.33
FF pH 5.7/270 min	4.78	472	16.90	0.32	5.10
H_2_O pH 6.5/1140 min	4.39	1640	26.00	0.84	5.02
H_2_O pH 6.5/2880 min	4.27	414	16.60	0.43	3.56
H_2_SO_4_ pH 2.0/150 min	2.75	775	25.70	0.22	9.50
H_2_SO_4_ pH 3.0/150 min	3.83	533	21.70	0.18	6.61
H_2_SO_4_ pH 3.8/150 min	3.26	311	17.10	0.15	4.60
H_2_SO_4_ pH 4.4/150 min	5.79	312	18.10	0.35	3.94
H_2_SO_4_ pH 4.4/150 min	2.42	576	15.30	0.22	5.57
H_2_C_2_O_4_ pH 3.1/15 min ^c)^	3.19	282	13.70	0.94	4.78
H_2_C_2_O_4_ pH 5.3/5740 min	16.30	3472	25.90	0.67	10.85

Notes:

^a^Measured on the surface.

^b^Extensive surface dissolution.

^c^Calcium oxalate was precipitating at the indent site.

#### Effect of pH

**Figure 4:**
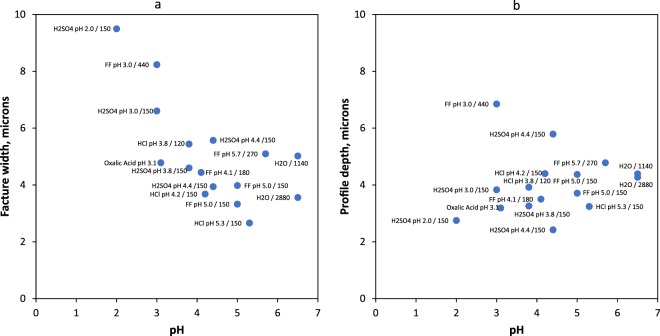
Surface morphology measurements by white light profilometry. The fluid pH and reaction times are listed for each sample; (**a**) Fracture width measured on the surface of the reacted samples vs. pH; and (**b**) Indent depth vs. pH.

As illustrated by our measurements, dissolution rate increased with decreasing solution pH. Surface morphology measurements agree with the dissolution rate measurements, as the measured fracture widths decrease with increasing pH (Fig. 4). The initial fractures generated by the nanoindenter were narrow, anastomosing features; surface dissolution caused an increase in the fracture widths as measured on the surface of the crystal. We anticipate that this surface dissolution took place at already opened fractures, and did not affect the stress field at the fracture tips. For each fluid type, the average fracture width increased with decreasing pH (Fig. 4). The trend is less clear with regards to the indent depth measurements (Fig. 4). With exception of the calcite sample reacted in synthetic hydrofracturing fluid at pH 3.0, it appears that in low pH fluids calcite surface was removed by dissolution surrounding the indentation site, resulting in shallower indentation depth with decreasing pH (Fig. 4).

#### Effect of exposure time

Total exposure times differed for different experiments. We observed that fracture widths measured on the surface increased with longer exposure times (Table 2). The largest fracture widths were observed in oxalic acid and fracking fluids for samples with the longest exposure times. The effect of exposure time had the opposite effect on indent depths compared to fluid pH: lower pH fluids caused a decrease in the indentation depths, but longer exposure times resulted in deepening of the indentation sites. We hypothesize that this is due to the damaged zone below the indent site being more susceptible to dissolution due to its high residual stress field and micro-cracks, compared to the undamaged calcite surface.

#### Changes in indentation morphology

**Figure 5:**
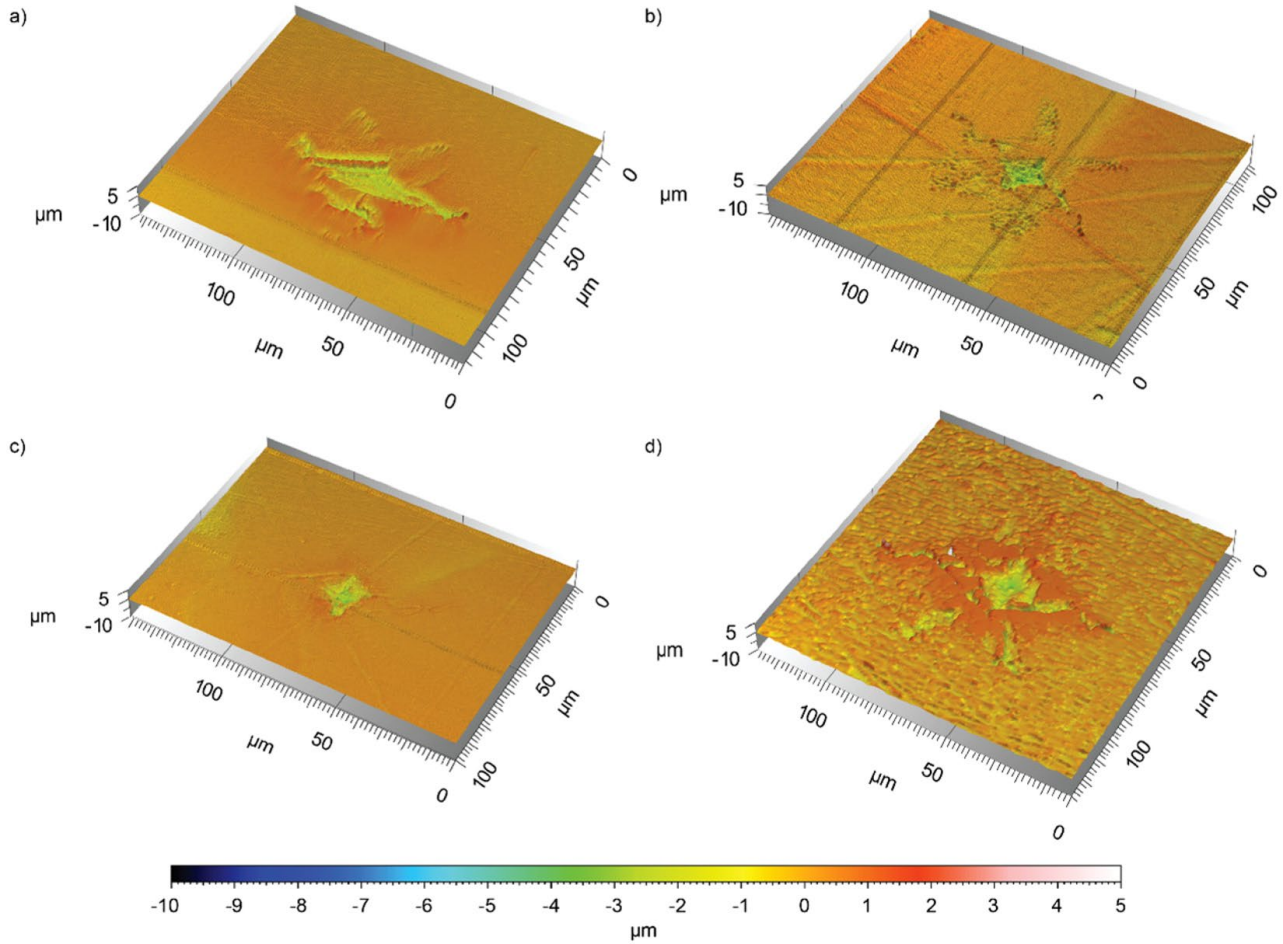
3D rendering of the reacted indented calcite surfaces: (**a**) Sample exposed to H_2_SO_4_ at pH 2.0 for 150 minutes; (**b**) Sample exposed HCl at pH 3.8 for 120 minutes; (**c**) Sample exposed to fracking fluid at pH 4.1 for 180 minutes; and (d) Sample exposed to DI H_2_O for 1140 minutes.

Nanoindentation in calcite results in a diamond shaped indent with narrow, anastomosing fractures growing diametrically away from one axis of the indentation site (see Methods section). The 3D renderings of a set of post-reaction calcite samples are shown in Fig. 5. For samples with low extent of surface dissolution, this basic geometry was maintained in the post-reaction samples (Fig. 5c); with increased dissolution, pitted and rough patches can be observed forming perpendicular to the indentation sites (Fig. 5b and d). With further dissolution, fractures growing away from the indent coalesce into shorter, wider fractures, the diamond shape of the indent is no longer distinguishable, and the patches forming perpendicular to the indentation sides become increasingly prominent (Fig. 5a).

### Surface charge considerations

**Figure 6:**
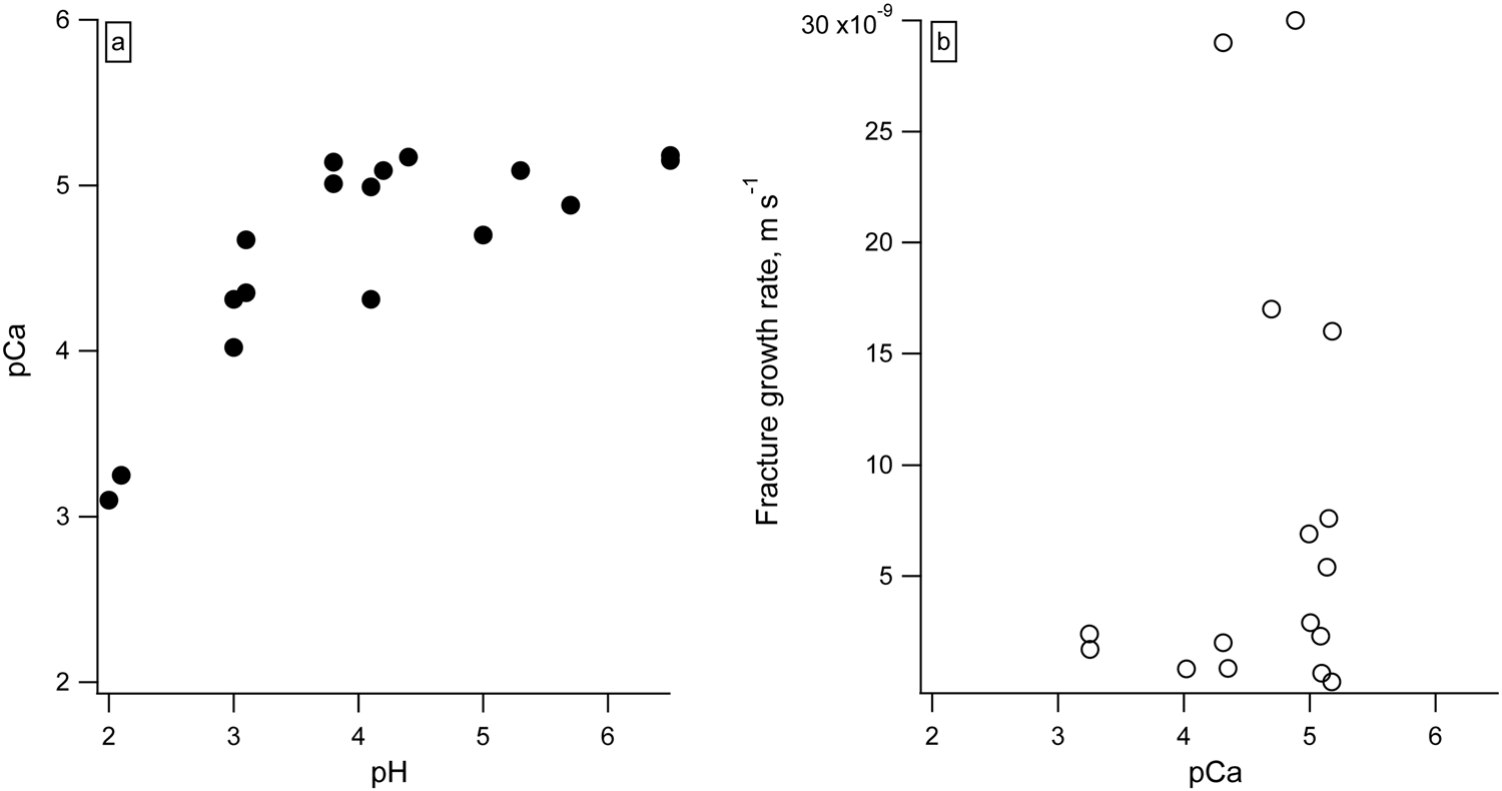
(**a**) Positive correlation observed between pCa and pH for all examined reactors; pCa = −log_10_[Ca^2+^]; (**b**) No correlation was observed between pCa (proxy for ξ-potential) and fracture propagation rates.

The ξ-potential of the calcite surface is controlled by the concentration of dissolved Ca^2+^, Mg^2+^, and CO_3_^2−^, not pH^26,33^. We compared the concentrations of dissolved Ca^2+^ in our reactors post exposure to estimate the expected magnitude of ξ-potential differences on calcite surfaces in different batch reactors. Previous studies on calcite have shown that the gradient dξ/dpCa is on the order of −8 to −15 mV/decade^33^, where pCa is reported as −log_10_[Ca^2+^]. The concentrations of Ca^2+^ measured after 180 minutes of reaction are shown in Table 3. The corresponding pCa values range from 3.10 to 5.18 and vary predictably with pH (Fig. 6a), in agreement with our dissolution rate measurements. We therefore estimate that the difference in the ξ-potential between pH ~2 and pH~5-6 reactors was on the order of −16 to −30 mV, and ξ-potential became more negative with decreasing pH. There was no correlation between the fracture growth rate and pH in any of the systems, except for the synthetic hydrofracturing fluids. In hydrofracturing fluids, the fracture propagation rate decreased with decreasing pH (see Supporting Information). When all fluid types are considered, there was no correlation between pCa (taken here as a proxy ξ-potential) and fracture growth rate (Fig. 6b); therefore, we propose that differences in the ξ-potential had a secondary effect on subcritical fracture. Previous work has demonstrated that Joffe’s effect, when a fracture tip is blunted due to dissolution, is not likely for calcite^21^. Our data illustrates that the propagation rate of subcritical fracture is dependent on the aqueous composition, and with increasing strength of the calcium-ligand complexes, the fracture propagation rate decreases. Therefore, we postulate that the primary chemical control on subcritical fracture growth in our experiments was chemical complexation at the fracture tip.

**Table 3:** Concentration of Ca^2+^ in all reactors after 180 minutes of reaction.

Reactor	Ca^2+^, M	pCa
HCl pH 2.1	5.57 × 10^−4^	3.25
HCl pH 3.1	4.48 × 10^−5^	4.35
HCl pH 3.8	7.31 × 10^−6^	5.14
HCl pH 4.2	8.19 × 10^−6^	5.09
FF pH 2.1	5.65 × 10^−4^	3.25
FF pH 3.0	9.58 × 10^−5^	4.02
FF pH 4.1	4.89 × 10^−5^	4.31
FF pH 5.0	2.01 × 10^−5^	4.70
FF pH 5.7	1.31 × 10^−5^	4.88
H_2_O pH 6.5	6.62 × 10^−6^	5.18
H_2_O pH 6.5	7.06 × 10^−6^	5.15
H_2_SO_4_ pH 2.0	7.98 × 10^−4^	3.10
H_2_SO_4_ pH 3.0	4.87 × 10^−5^	4.31
H_2_SO_4_ pH 3.8	9.88 × 10^−6^	5.01
H_2_SO_4_ pH 4.4	6.70 × 10^−6^	5.17
H_2_C_2_O_4_ pH 3.1	2.15 × 10^−5^	4.67
H_2_C_2_O_4_ pH 4.1	1.01 × 10^−5^	4.99
H_2_C_2_O_4_ pH 5.3	8.05 × 10^−6^	5.09

### Conceptual model and environmental implications

Previous studies illustrated that when calcite is fractured, the freshly cleaved surface is highly reactive with water molecules present, even at low water activity (e.g., in the air), forming >Ca—OH and >CO_3_—H surface hydrolysis products^26,27^. Our observations indicate that the fracture growth rate decreased with increasing favorability for the Ca-ligand complex (e.g., K_β_ for CaCO_3_ is 10^−7.128^; K_β_ for CaCl^+^ is 10^0.7^; and K_β_ for CaSO_4_ is 10^2.32^). We propose that the following exchange reactions occur at the calcite surface inside the fracture tip:

R1 $$ > {\rm{Ca}}-{\rm{OH}}+{{\rm{H}}}_{2}{{\rm{CO}}}_{3}\to  > {\rm{Ca}}-{{\rm{CO}}}_{3}^{-}+{{\rm{H}}}^{+}+{{\rm{H}}}_{2}{\rm{O}}$$

R2 $$ > {\rm{C}}{\rm{a}}-{\rm{O}}{\rm{H}}+{\rm{H}}{\rm{C}}{\rm{l}}\to  > {\rm{C}}{\rm{a}}-{\rm{C}}{\rm{l}}+{{\rm{H}}}_{2}{\rm{O}}$$

R3 $$ > {\rm{C}}{\rm{a}}-{\rm{O}}{\rm{H}}+{{\rm{H}}}_{2}{{\rm{S}}{\rm{O}}}_{4}\to  > {\rm{C}}{\rm{a}}-{{{\rm{S}}{\rm{O}}}_{4}}^{-}+{{\rm{H}}}^{+}+{{\rm{H}}}_{2}{\rm{O}}$$

**Figure 7:**
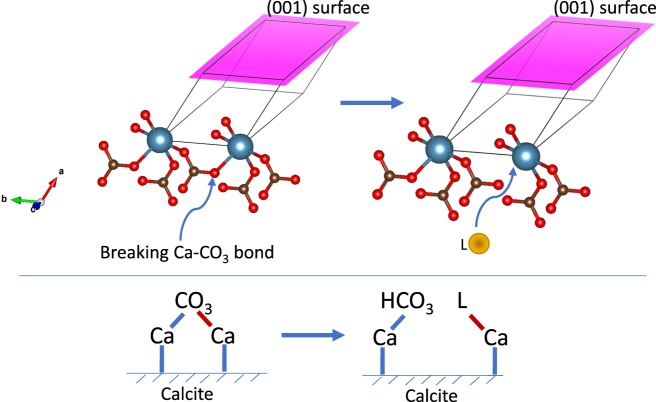
Proposed schematic for the complexation reactions at the fracture tip; L = ligand.

We hypothesize that with increasing strength of the Ca-ligand complex it becomes harder for H_2_O molecules to break Ca—CO_3_ bonds at the fracture tip. The proposed mechanism is shown in Fig. 7. This local re-structuring of the calcite surface likely leads to the change in the sum of the elastic potential energy of chemical bonds at the fracture tip U_E_.

In natural environments the formation brine chemistry can vary widely, from carbonate-, to chloride-, to sulfate-rich endmembers. Changes is the formation brine chemistry due to fluid injections, or due to the propagation of faults through a reservoir, may result in strengthening or weakening of carbonate cements. If these physical changes result in the introduction of strongly-complexing ligands (e.g. sulfate), or increasing salt concentrations, carbonate cements would become stronger. Contrary, if formation brine chemistry shifts towards weaker-complexing ligands (e.g. bicarbonate), mechanical weakening and potentially fracturing could be expected.

## Conclusions

We used a novel experimental technique to measure the growth of subcritical fractures in calcite single crystals *in situ* (in liquid). We identified chemical controls on subcritical fracture by testing a range of aqueous chemistries, including de-ionized water, dilute hydrochloric, sulfuric, and oxalic acids, and synthetic hydrofracturing fluid. Each fluid’s pH varied from 2 to 5–6, and exposure time was up to 240 minutes (4 hours). We tested the effects of (i) chemical ligands present in the solution, (ii) mineral dissolution rate and (iii) pH, on the propagation rate and geometry of subcritical fracture. Our findings can be summarized as follows:

1. The propagation rate of subcritical fracture measured *in situ* varied from 1.6 × 10^−8^ m s^−1^ to 2.4 × 10^−10^ m s^−1^, and fracture propagation rate depended on the aqueous ligand present.
2. In synthetic hydrofracturing fluid, the propagation rate of fracture was slower as pH decreased; however, no relationship between fracture growth rate and pH was observed in dilute hydrochloric, oxalic, and sulfuric acid solutions.
3. In agreement with the previous studies^6,21^, there was no correlation between the dissolution rate of calcite and subcritical fracture growth.
4. Post-reaction sample examination showed that the fracture width, measured on the surface of the sample, increased with decreasing pH values, in agreement with enhanced calcite surface dissolution with decreasing pH.
5. The estimated difference in the ξ-potential of the calcite surface between different reactors was on the order of −16 to −30 mV; however, we observed no correlation between the pCa values (used as a proxy for ξ-potential) and the kinetics of fracture growth.

We propose that favorable Ca-anion complexation “healed” the mineral surface at the fracture tip, shielding calcium-carbonate (Ca—CO_3_) bonds on calcite surfaces within the fracture tip. This complexation prevented further Ca—CO_3_ bond breakage and decreased the fracture growth rate.

## Materials and Methods

### Sample preparation, *in situ* fracture and dissolution rate measurements

**Figure 8:**
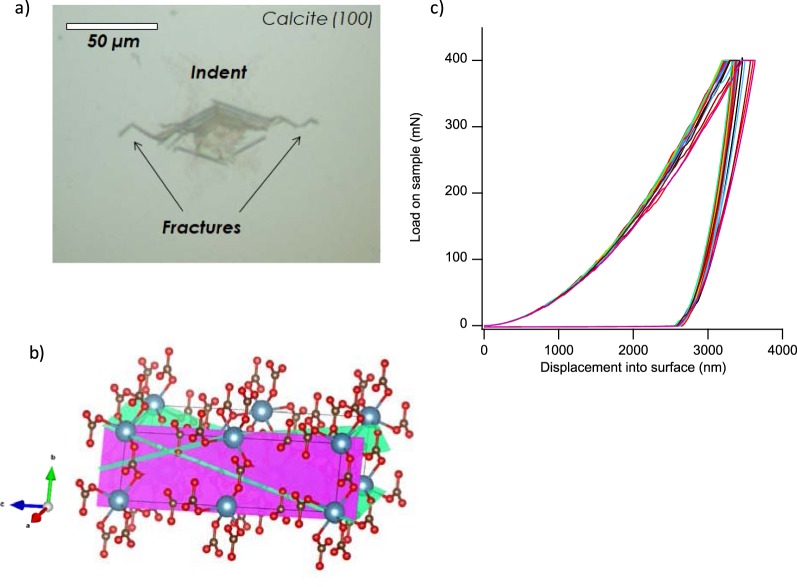
(**a**) Microphotograph of indented calcite (100) surface with fractures propagating form the indent site; (**b**) Calcite structure, showing (100) crystallographic plane with magenta surface, and rhombohedral cleavage planes with green intersecting surfaces. Structure visualized using VESTA 3^35^; (**c**) The load-displacement response for 25 Vickers indents into calcite show the repeatability of the indent.

Single crystal calcite CaCO_3_ samples cut along the (100) crystallographic surface were purchased from MTI corporation (http://www.mtixtl.com/CCO-a-101010-S1.aspx). The calcite (100) surface was indented under dry conditions using a Vickers indenter tip to a maximum load of 400 mN, resulting in initial fractures propagating from the indent site (Fig. 8a and b). The size of the indent with initial fractures was ~ 65–85 µm. The crystal was cut into 2 mm by 2 mm pieces, with each piece containing one indent. The loading-unloading curves (Fig. 8c) confirm the repeatability of the indentation process. This, in combination with similar initial crack length, confirms that all indents had similar residual stress at the fracture tips prior to the fracture growth experiments.

The individual indented samples were submerged in 2-3 mL of aqueous fluids with differing compositions and pH (Table 4). During the exposure to aqueous solutions, fractures grew from the indent site along cleavage planes in calcite, which we monitored *in situ* using a Nikon Eclipse 80i optical microscope in combination with a SPOT 7.2 camera. Images were taken before the experiment (dry sample), following by imaging on average after 1, 2, 3, 4, 5, 8, 10, 12, 15, 20, 25, 30, 35, 45, 60, 75, 90, 120, and 180 minutes of reaction. Each experiment lasted for up to four hours. Fracture length was measured using ImageJ analysis software^34^.

**Table 4:** Aqueous fluids used in *in situ* calcite fracturing and dissolution rate measurements.

Fluid	Composition	pH
De-ionized (DI) H_2_O	DI H_2_O	6.5
Hydrochloric acid (HCl)	DI H_2_O, HCl	5.3; 4.2; 3.8; 3.1; 2.1
Sulfuric acid (H_2_SO_4_)	DI H_2_O, H_2_SO_4_	4.4; 4.5; 3.8; 3.0; 2.0; 1.4
Oxalic acid (H_2_C_2_O_4_)	DI H_2_O, H_2_C_2_O_4_	5.3; 4.1; 3.9
Hydrofracturing fluid	DI H_2_O, 0.01 vol.% polyacrylamide; 0.05 vol.% sodium polyacrylate; 0.1 vol.% sodium chloride; 0.02 vol.% methanol; 0.01 vol.% hydrochloric acid; 0.007 vol.% tetrakis(hydroxymethyl)phosphonium sulfate	5.7; 5.0; 4.1; 3.0; 2.1

To test whether the dissolution rate of calcite influenced the rate of fracture propagation, we measured dissolution rate in each of the tested aqueous solutions. We used a 1 cm^2^ calcite crystal (100) surface, which was polished before each dissolution experiment, while its non-polished surface was sealed with QuickStick binder to prevent dissolution. For polishing, a thin layer of QuickStick was melted on a heated steel puck, the calcite was mounted on it and allowed to cool. The crystal was polished with 0.5 µm diamond paste by hand lapping on a Microcloth pad for 1 to 5 minutes, then rinsed with acetone and de-ionized Milli-Q water with a resistivity of 18 MΩ (referred to as “DI H_2_O” in the text). Then the surface was polished for 5 minutes with colloidal silica at a pH of 11.5, adjusted using concentrated NaOH solution. When the NaOH solution began to dry on the pad, NaOH at pH 10 was added, and the crystal was polished for an additional 5 minutes. Finally, DI H_2_O was added onto the polishing pad, and the crystal was polished for an additional 2 minutes. The crystal was rinsed with DI H_2_O, and the remaining water was wicked away with lint-free paper. The initial ratio of mineral surface area to fluid volume was kept constant between the fracture growth experiments and dissolution rate measurements. The 1 cm^2^ crystal was placed in a Teflon beaker with 50 mL of aqueous fluid, slowly agitated on a shaker table, and samples were collected throughout the 3 hour-experiment. Aqueous samples were preserved with 6 N ultrapure HNO_3_, and aqueous Ca concentration was quantified using inductively coupled plasma mass spectrometer (ICP-MS) equipped with a collision-reaction cell. Calcium was quantified in the reaction mode using an ammonia gas flow of 0.6 mL min^−1^. Several dissolution experiments were repeated in triplicate. To test the integrity of the QuickStick coating on the back of the sample, 0.5 g of crushed QuickStick was soaked for 24 hours in every tested aqueous solution. No calcium was detected in the leachate prepared by soaking, therefore demonstrating that no calcium leached from the QuickStick coating on the back side of the sample.

### *Ex situ* fracture geometry analysis by white light profilometry and confocal Raman spectrometry

**Figure 9:**
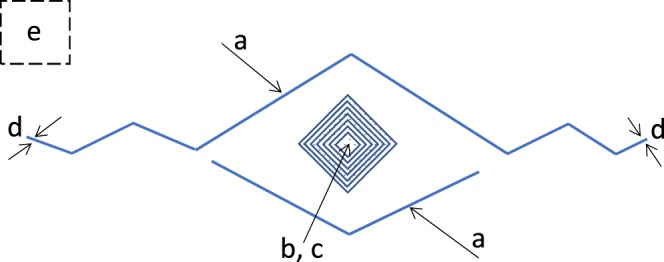
Schematic for the white light profilometry measurements: (**a**) Indent width; (**b**) Profile depth; (**c**) Indent volume; (**d**) Fracture tip with (an average for two fractures is reported); and (**e**) Surface roughness.

After fracture growth measurements, calcite samples were taken out of the fluid and the remaining fluid was immediately wicked away using lint-free paper. Surface heights were measured to characterize fracture geometry, surface roughness, and calcite dissolution at the indentation site. Surface height was measured using a non-contact white LED light surface profilometer Nanovea ST-400 with a motorized x-y stage. All measurements were made with an optical pen with a measurement range of 110 μm and a maximum linearity error of 0.025 μm, capable of resolving surfaces with a surface angle of less than 43 degrees to the horizontal. The scan area was 400 μm by 300 μm, and scans were performed centered around the nanoindentation with a measurement spacing of 0.1 μm. The scans were processed using Mountains 3D analysis software. Raw images were leveled, flattened, and cropped around the indentation site, as needed. Profile depth and indent volume were calculated using a 3D hole measurement tool. The schematic for surface measurements is shown in Fig. 9. Indent width was measured perpendicular to one of the sides of the indent. Fracture width was measured close to the visible end of each fracture, just before the visible rounded portion, and reported as an average of both fracture ends. Surface roughness measurements were made away from the indentation site and are reported as the arithmetical mean height of the surface.

Raman spectra were acquired using WITec alpha300 spectrometer using 532 nm excitation wavelength. X-Z cross-sections were acquired from two areas at control (unreacted) and a sample reacted in synthetic hydrofracturing fluid at pH 5.0. For control sample, two cross-sections were obtained from 9 by 9 microns (90 × 90 pixels) and 2 by 4 (20 × 40 pixels) areas. For the reacted sample, two regions from 13 by 20 microns (130 by 200 pixels) were obtained.

### Geochemical Modeling

The Geochemist’s Workbench (GWB) software^32^ was used to calculate aqueous speciation during the reaction between calcite and tested aqueous solutions (React module). Reaction path modeling was used to predict aqueous speciation for Ca, released from calcite, as a function of reaction progress. The dissolution was modeled as a kinetic process, with the dissolution rate constants specified for each case with values measured in our experiments. The DI H_2_O (pH 6.5), and acidic solutions at pH 4, including HCl and H_2_SO_4_ were modeled. Standard thermodynamic database *thermo.dat* from the Lawrence Livermore National Laboratory (LLNL) included in the GWB package was used.

## Electronic supplementary material

Supplementary Information

## Data Availability

All data generated or analyzed during this study are included in this published article (and its Supplementary Information files).
