# Supplementary material for: Chemical controls on the propagation rate of fracture in calcite
Source: Sci Rep. 2018 Nov 7;8:16465. doi: 10.1038/s41598-018-34355-1 (PMC6220247; doi:10.1038/s41598-018-34355-1)
Supplement: Supplementary file 1 — Supplementary Information [file 41598_2018_34355_MOESM1_ESM.docx]

Supporting Information

**Chemical controls on the propagation rate of fracture in calcite**

A.G. Ilgen*^1^, W.M. Mook^2^, A.B. Tigges^1^, R.C. Choens^3^, K. Artyushkova^4^, and K.L. Jungjohann^2^

1. Sandia National Laboratories, Geochemistry Department, 1515 Eubank SE Mailstop 0754, Albuquerque, NM 87185-0754, United States
2. Sandia National Laboratories, Nanosystems Synthesis/Analysis Department, Center for Integrated Nanotechnologies, Albuquerque, NM, United States
3. Sandia National Laboratories, Geomechanics Department, 1515 Eubank SE Mailstop 0750, Albuquerque, NM 87185-0750, United States
4. University of New Mexico, Advanced Materials Laboratory, Albuquerque, NM, United States

*Corresponding author. E-mail [agilgen@sandia.gov](mailto:agilgen@sandia.gov)

Pages: 6

Tables: 1

Figures: 4

**S1. Dissolution rate of calcite**

We measured the dissolution rates for calcite (100) surfaces, in the same aqueous solutions that were used in the fracture propagation experiments. Measured dissolution rates are shown in Table 2 (main text). Our measured rates agree with published dissolution trends: dissolution rate is constant for pH values of 5 and 6, and dissolution rate sharply increase with decreasing pH (Figure S1). The measured dissolution rates at pH values between 2 and 4 are about one order of magnitude lower than the rates summarized in the review by Arvidson et al., 2003 (Figure S1). [^1^](#_ENREF_1) We propose that our measured rates are slower, because our measurements were performed on single crystal (100) surfaces and on a stationary sample, with slight agitation. A single crystal surface has fewer defect sites compared to the crushed powder form, and as a result, slower dissolution rate. In agreement with previous work [^2^](#_ENREF_2), dissolution rates are weakly affected by the variation in solution ligand.


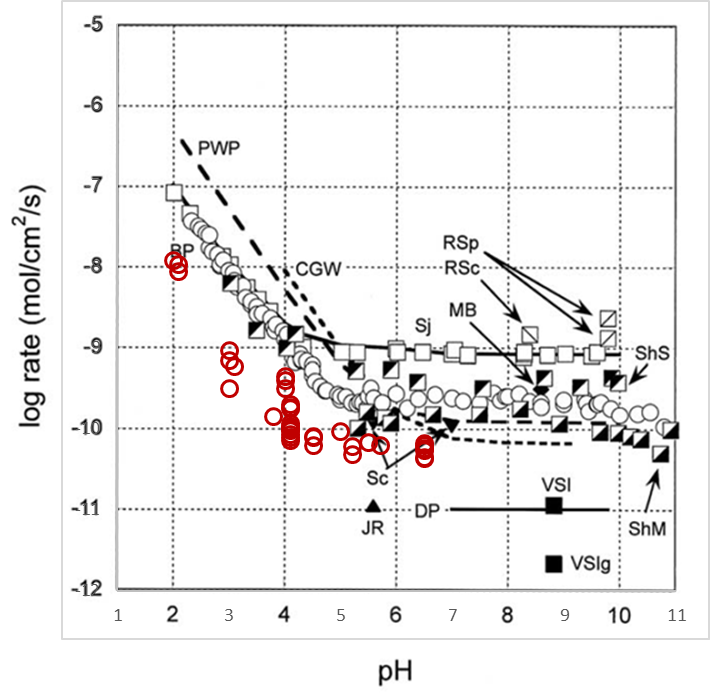


**Figure S1.** Calcite dissolution rates measured in our experiments vs. Arvidson et al., 2003 summary of the previously-measured dissolution rates.[^1^](#_ENREF_1) Our measurements are shown as red empty circles.

**S2. In situ fracture growth**

The fracture propagation rates for calcite in dilute hydrochloric, sulfuric, oxalic acids, and in synthetic hydrofracturing fluid are shown in Figure 1 (main text), and summarized in Table 1 (main text). Our measured fracture propagation rates did not correlate with calcite dissolution rates, zeta-potential, or fluid pH in any systems (Figure S2) except for the synthetic hydrofracturing fluid case. A positive correlation between fracture propagation rate and pH was observed for experiments performed in synthetic hydrofracturing fluid (Figure S2). In hydrofracturing fluids, the propagation rate of fracture was slower as pH decreased.


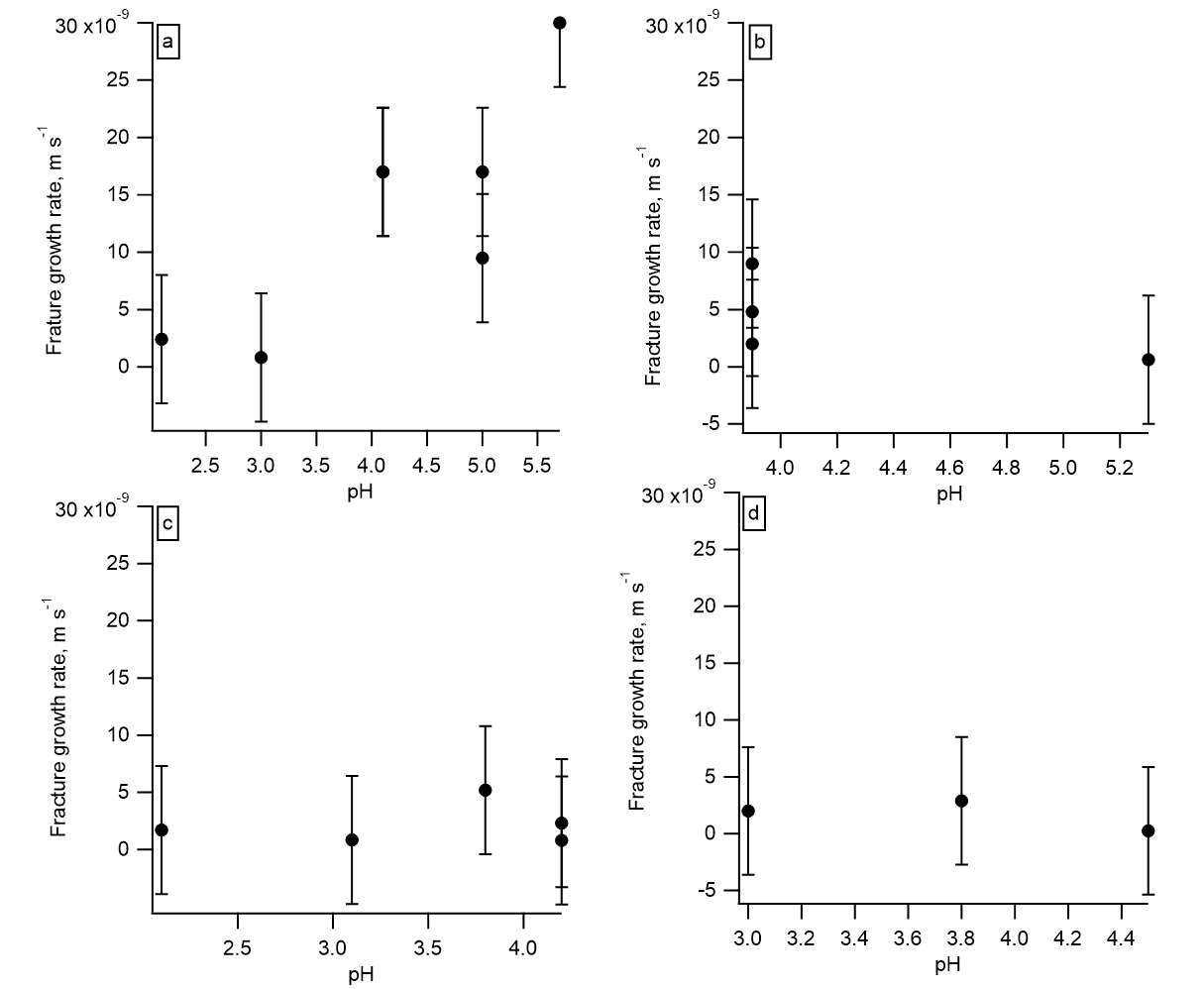


**Figure S2.** (a) Fracture propagation rate vs. pH in synthetic hydrofracturing fluid, (b) in dilute oxalic, (c) in dilute hydrochloric, and (d) in dilute sulfuric acids. No correlation is observed, except for synthetic hydrofracturing fluid.

**S3. Aqueous speciation of calcium calculated for 0.01-4M NaCl solutions**

Using Geochemist’s Workbench, [^3^](#_ENREF_3) we calculated aqueous speciation in the solutions used in double torsion experiments by Rostom et al., 2012. In these experiments, strengthening was observed with increasing NaCl concentration, with strengthening in the 0.8M-4M NaCl solutions, when compared to 0.01M NaCl.[^4^](#_ENREF_4) Our aqueous speciation calculations show, that similar to our systems, the most common aqueous species was Ca^2+^, and the second most abundant species was CaCl^+^. Our modeling results are shown in Figure S3. We observed that the [Ca^2+^]/[CaCl^+^] ratio in the 0.01M NaCl is much lower, indicating a lower predicted abundance of the CaCl^+^ complex, compared to the rest of the NaCl solutions with NaCl concentration ranging from 0.1M to 4M (Figure S4). We propose that strengthening was observed due to increasing Ca-chloride complexation at the fracture tip with increasing NaCl concentration.

**Figure S3.** Calculated aqueous speciation in the reactors tested by double torsion reported by Rostom et al., 2012. The [Ca^2+^]/[CaCl^+^] ratio for 0.01M is larger, compared to the rest of the solutions with 0.1 M up to 4M NaCl solutions.

**S4. Confocal Raman Imaging**

Raman spectra were acquired using WITec alpha300 spectrometer using 532 nm excitation wavelength. X-Z cross-sections were acquired from two areas on control (unreacted) sample and a sample reacted in synthetic hydrofracturing fluid at pH 5.0. For the control sample, two cross-sections were obtained from 9 by 9 microns (90 x 90 pixels) and 2 by 4 microns (20 x 40 pixels) areas (Figure S4a). For the reacted sample, two regions from 13 by 20 microns (130 by 200 pixels) were obtained (Figure S4b). Through analysis of multi-spectral Raman images, two color-coded images were extracted with red representing voids in the crystal, while blue represents solid matter. Figure S4 shows optical images with lines marking the locations of cross-sections and red-blue overlays showing the penetration of the cracks into the crystal. The width of the crack in the control sample is on the order of 0.5 microns, measured at 1-2-micron depth, while that for the reacted sample is between 2 to 5 microns at the same depth. Due to scattering of light coming through the material, there is a loss of signal at depths greater than 4-5 microns.


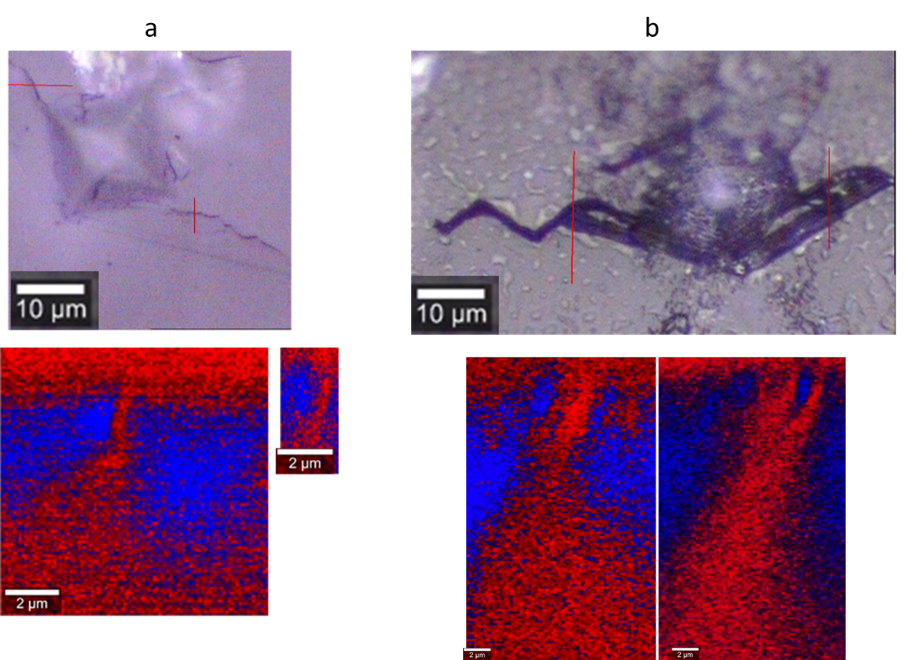


**Figure S4.** Optical images with lines marking the locations of cross-sections and red-blue overlays showing the penetration of the crack into the crystal: (a) Control sample (not reacted), and (b) Sample reacted in synthetic hydrofracturing fluid at pH 5.0. Red color represents voids in the crystal, and blue color represents solid matter.

**S5. Fracture Toughness calculations**

The initial cracks on calcite (100) surface were created following indentation with the Vickers tip to a maximum load of 400 mN. These cracks were in equilibrium with the residual stress around the crack tip after samples were unloaded. [^5^](#_ENREF_5) We observed that these fractured began to grow upon exposure to liquids, which means a decrease in fracture toughness. To estimate the decrease in fracture toughness, we used the method described by Lawn and Cook, 2012. The fracture toughness can be estimated from [^5^](#_ENREF_5):

$\frac{P}{c^{3/2}}=\frac{1}{\xi}\times\left( \frac{H}{E} \right)^{1/2}\times T$

Where *P* is the maximum load (400 mN), *c* is fracture length, measured form the center of the indent, *H* is hardness, *E* is indentation modulus, ξ is the dimensionless constant (0.016 for the Vickers tip), and *T* is fracture toughness. Using our indentation data, we determined that *H* was 3.123 GPa, and *E* was 73.961 GPa. The calculated fracture toughness is shown in Table S1.

**Table S1.** Calculated fracture toughness before and after exposure to aqueous solutions. The uncertainty in fracture toughness value is ±0.01-0.02 (shown in parenthesis with each calculated T value), calculated at 2ϭ (95% confidence level).

| **Reactor** | **pH** | **Fracture length (initial),**  **microns** | **Fracture length (final), microns** | ***c* ^a)^ (initial), microns** | ***c* ^a)^ (final), microns** | **T ^b)^ (initial), MPa m^1/2^** | **T ^b)^ (final), MPa m^1/2^** |
| --- | --- | --- | --- | --- | --- | --- | --- |
| DI H_2_O | 6.5 | 89.2 | 98.8 | 44.6 | 49.4 | 0.10±0.01 | 0.09±0.01 |
| DI H_2_O | 6.5 | 82.0 | 99.1 | 41.0 | 49.6 | 0.12±0.01 | 0.09±0.01 |
| DI H_2_O | 6.5 | 73.6 | 93.4 | 36.8 | 46.7 | 0.14±0.02 | 0.10±0.01 |
| FF | 4.1 | 83.8 | 96.0 | 41.9 | 48.0 | 0.11±0.01 | 0.09±0.01 |
| HCl | 3.8 | 67.5 | 84.5 | 33.8 | 42.3 | 0.16±0.02 | 0.11±0.01 |
| H_2_SO_4_ | 3.8 | 73.0 | 75.0 | 36.5 | 37.5 | 0.14±0.02 | 0.14±0.02 |
| C_2_H_2_O_4_ | 4.1 | 72.7 | 99.0 | 36.4 | 49.5 | 0.14±0.02 | 0.09±0.01 |

Notes:

1. C is calculated as ½ of the full fracture length
2. T is fracture toughness

**S6. References**

1 Arvidson, R. S., Ertan, I. E., Amonette, J. E. & Luttge, A. Variation in calcite dissolution rates: A fundamental problem? *Geochim. Cosmochim. Acta* **67**, 1623-1634 (2003).

2 Pokrovsky, O. S., Golubev, S. & Jordan, G. Effect of organic and inorganic ligands on calcite and magnesite dissolution rates at 60 C and 30 atm pCO2. *Chem. Geol.* **265**, 33-43 (2009).

3 Bethke, C. *Geochemical and biogeochemical reaction modeling*. Vol. 543 (Cambridge University Press Cambridge, UK, 2008).

4 Rostom, F., Røyne, A., Dysthe, D. K. & Renard, F. Effect of fluid salinity on subcritical crack propagation in calcite. *Tectonophysics* **583**, 68-75 (2013).

5 Lawn, B. R. & Cook, R. F. Probing material properties with sharp indenters: a retrospective. *Journal of Materials Science* **47**, 1-22 (2012).
